# Photochemistry and the role of light during the submerged photosynthesis of zinc oxide nanorods

**DOI:** 10.1038/s41598-017-18572-8

**Published:** 2018-01-09

**Authors:** Lihua Zhang, Melbert Jeem, Kazumasa Okamoto, Seiichi Watanabe

**Affiliations:** 1 Faculty of Engineering, Hokkaido University, N13, W8, Kita-ku, Sapporo, 060-8628 Japan; 2 Graduate School of Engineering, Hokkaido University, N13, W8, Kita-ku, Sapporo, 060-8628 Japan

## Abstract

Recently, metal oxide nanocrystallites have been synthesized through a new pathway, i.e., the submerged photosynthesis of crystallites (SPSC), and flower-like ZnO nanostructures have been successfully fabricated via this method. However, the photochemical reactions involved in the SPSC process and especially the role of light are still unclear. In the present work, we discuss the reaction mechanism for SPSC-fabricated ZnO nanostructures in detail and clarify the role of light in SPSC. The results show that both photoinduced reactions and hydrothermal reactions are involved in the SPSC process. The former produces OH radicals, which is the main source of OH^**−**^ at the ZnO crystal tips, whereas the latter generates ZnO. Although ZnO nanocrystals can be obtained under both UV irradiation and dark conditions with the addition of thermal energy, light promotes ZnO growth and lowers the water pH to neutral, whereas thermal energy promotes ZnO corrosion and increases the water pH under dark conditions. The study concludes that the role of light in the submerged photosynthesis of crystallites process is to enhance ZnO apical growth at relatively lower temperature by preventing the pH of water from increasing, revealing the environmentally benign characteristics of the present process.

## Introduction

Metal oxides are one of the most widely investigated inorganic substances because they are ubiquitous in nature and frequently used in technological applications. The wide range of nanoscale forms of these materials, called metal oxide nanocrystals (NCs) that can be formed as nanowires, nanotubes, and nanorods, have gained much attention in recent years owing to their anticipated properties and application in different areas, such as photoelectron devices, sensors, catalysts, and photovoltaic devices^1–8^. Among these metal oxide NCs, zinc oxide is one of the most important natural *n*-type semiconductors with a direct wide band gap in the near-UV spectral region (3.36 eV at room temperature)^9–11^. ZnO is a promising material for fabricating devices for optoelectronics and photonics applications^12–14^. The optical and electrical properties of ZnO are affected by many factors, such as the structure^15,16^, size^17^, shape^18,19^, and defect concentration^19,20^, which makes ZnO a very important and interesting subject to examine the controlled growth of novel materials.

In a previous study, Jeem *et al*.^21^ reported a new pathway for the synthesis of a variety of metal oxide NCs via submerged illumination in water, called the submerged photosynthesis of crystallites (SPSC). This method is completely different from typical synthetic methods for nanoparticles, such as the hydrothermal method^22–24^, solvothermal synthesis^25,26^, and chemical vapor deposition (CVD)^27–29^. In the SPSC method, the initial metal is surface treated by a submerged liquid plasma process, which creates a metal nano oxide semiconducting layer with surface protrusions. After that, the growth of metal oxide NCs is assisted by a ‘photosynthesis’ reaction, where the metal surface is irradiated with ultraviolet (UV) light in water^21^. Thus, the SPSC process requires only light and water and does not require the incorporation of impurity precursors. Moreover, this method is applicable at low temperature and at atmospheric pressure, producing only hydrogen gas as the by-product. These characteristics give rise to the potential application of SPSC as a green technology for metal oxide NC synthesis.

At present, flower-like NCs of zinc oxide^21,30^ and cupric oxide^31^ have been successfully synthesized using the SPSC method. Previous work^21,30,31^ has discussed the reactions in the SPSC process and shown that the process is photocatalytic, accompanied by hydroxyl radical generation via water splitting. The shape of ZnO nanorods (NRs), from tapered to capped-end, could be controlled by the SPSC process, and oxygen vacancy point defects near the tip-edge of the NRs were found to be opto-electrical hotspots for light-driven formation^30^. As is well known, Zn metal itself can react with water to form Zn^2+^ ions and OH^−^ with H_2_ gas generation even in dark conditions, according to the Pourbaix diagram of the Zn-H_2_O system^32^, and ZnO precipitates in alkaline aqueous environments. Therefore, ZnO NCs can also be obtained from zinc and water without illumination in the dark by controlling the pH of the water. Comparing the SPSC process with the reactions under dark conditions raises the following question: what is the role of light in the SPSC of ZnO NCs? However, this question has not been addressed, and the photochemical reactions that occur in the complicated hydrothermal process are still unclear. The present study of the photochemistry of SPSC for ZnO NC fabrication on a zinc surface was conducted by irradiation with UV light in ultrapure water, and the photochemical reaction mechanism was elucidated. Furthermore, the reactions involved in the SPSC process and under dark conditions are analyzed by monitoring the pH and temperature changes of the water, based on which a photoinduced enhancement factor is introduced to discuss the role of light in the SPSC process.

## Results and Discussion

**Figure 1:**
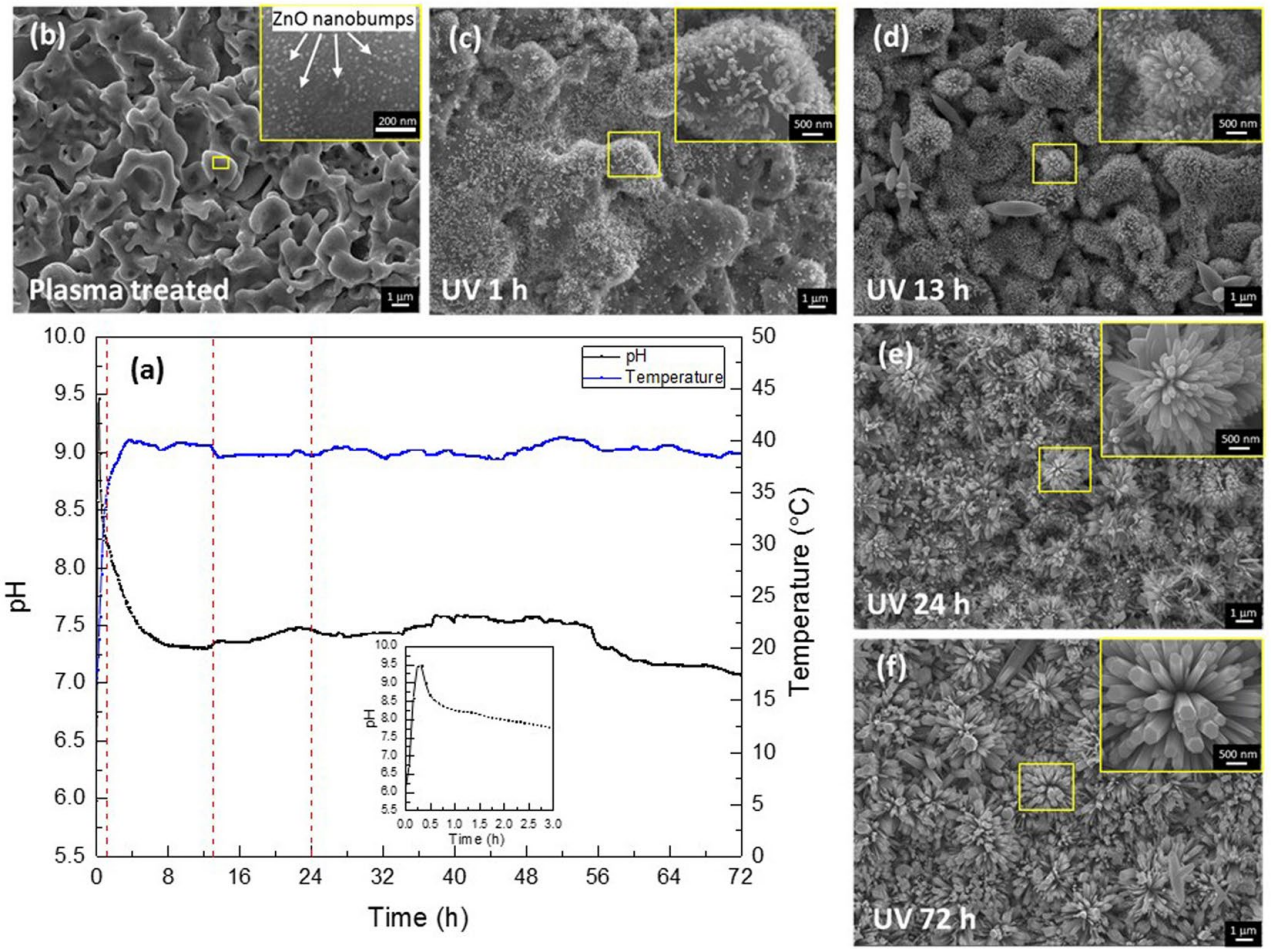
(**a**) Time dependence of the pH and temperature of ultrapure water during the UV-irradiation SPSC experiment. The inset is a magnified view of the pH change over a period of 3 h. (**b**) SEM image of the surface of plasma-treated Zn plate. (**c–f**) SEM images of the surface of the Zn plate after different UV irradiation times (1 h, 13 h, 24 h, and 72 h).

Figure 1a shows the time dependence of pH and temperature during a 72-h UV-irradiation SPSC experiment. The water temperature increased rapidly from room temperature (18 °C) to 39 °C during the first 3 h of irradiation due to energy from the UV light. After that, the temperature became relatively stable (39 ± 1°C). The pH of the water exhibited a sharp peak in the initial 4 h, after which the pH decreased to 7.0–7.6. To confirm the existence of the first sharp peak, the change in the water pH with the untreated Zn plate under UV-irradiation SPSC conditions and dark conditions was measured, and the pH curves showed similar trends. Therefore, the sharp peak was not caused by the alkaline electrolyte K_2_CO_3_ solution used in the plasma pretreatment process nor by UV irradiation. This result indicates that Zn undergoes dissolution in water. According to the Zn/H_2_O Pourbaix diagram^32^, the reactions involved in the sharp pH peak are considered as Zn corrosion reactions, which will be discussed in detail later.

Figure 1b shows the SEM images of the surface of the Zn plate after plasma pretreatment. Distributed over the surface are micrometer-size protrusions and numerous nanobumps. The nanobumps act as seeds for ZnO NCs and are approximately 10–20 nm in size^21^. After 1 h of UV irradiation, the nanobumps grow into ZnO nanorods (NRs) with lengths of approximately 200–400 nm (Fig. 1c, Figure S1 in the Supplementary information). The NRs show apical growth with increased irradiation time (Fig. 1d–f), and flower-like ZnO nanostructures were observed when the UV irradiation time was longer than 24 h.

**Figure 2:**
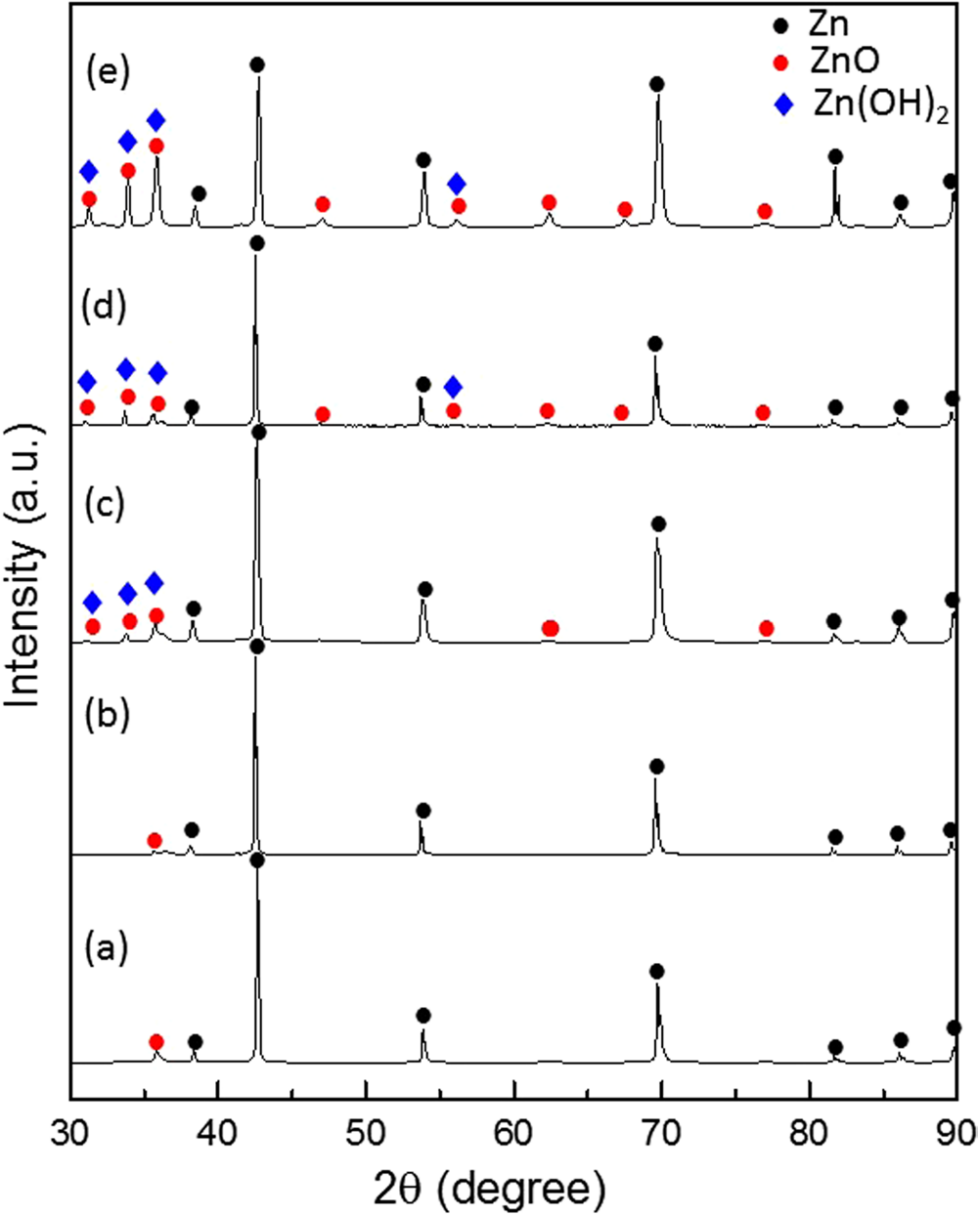
X-ray diffraction patterns of the Zn specimens after (**a**) plasma pretreatment, (**b**) 1 h of UV irradiation, (**c**) 13 h of UV irradiation, (**d**) 24 h of UV irradiation, and (**e**) 72 h of UV irradiation.

**Figure 3:**
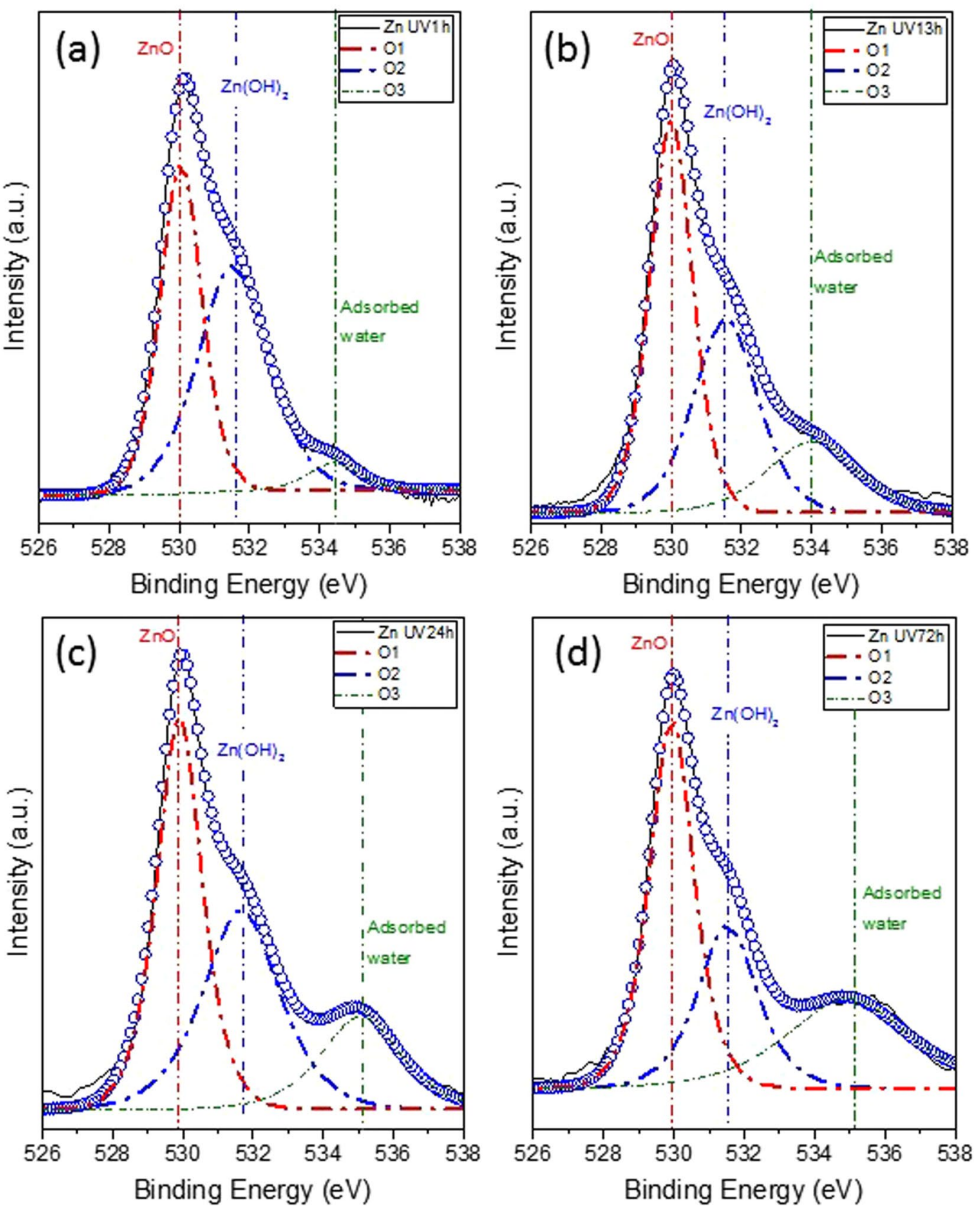
XPS spectra of the O 1 s peak of the Zn specimens after UV irradiation for different times: (**a**) 1 h, (**b**) 13 h, (**c**) 24 h, and (**d**) 72 h.

Figure 2 shows the XRD patterns of the specimens after surface pretreatment (Fig. 2a) and after different UV irradiation times (Fig. 2b–e). After plasma pretreatment, wurtzite structured ZnO (Zincite, JCPDS 5-0664) was observed, which confirmed that ZnO forms on the surface of the Zn plate (Zinc, JCPDS 4-0831) via the plasma pretreatment process. After UV irradiation, ZnO peaks were enhanced, and the peak intensity increased with irradiation time, which is consistent with the SEM images shown in Fig. 1c–f. When the UV irradiation time was 72 h, the highest ZnO ratio was obtained. Moreover, Zn(OH)_2_ (Zinc Hydroxide, JCPDS 48-1066) peaks were also detected by XRD. As the peak position of Zn(OH)_2_ is very close to that of ZnO^33^, XPS spectral analysis was utilized to confirm the formation of Zn(OH)_2_. Figure 3 displays the XPS O 1 s spectra of the specimens after different UV irradiation times. The Shirley method was performed to subtract the background. Three Gaussian-Lorentzian fitting peaks, denoted O1, O2, and O3, were used to fit the experimental data. The O1 peak located at the lower binding energy of 529.9–530.1 eV is assigned to O^2−^ ions involved in Zn-O bonding of the wurtzite structure of ZnO^34^. The O2 peak located at 531.1–531.8 eV is typically assigned to loosely bound oxygen on the surface OH groups^35^. The O3 peak is attributed to adsorbed water^36^. Therefore, the O2 peak proved that Zn(OH)_2_ is present on the surface of the specimens as a result of the SPSC process.

**Figure 4:**
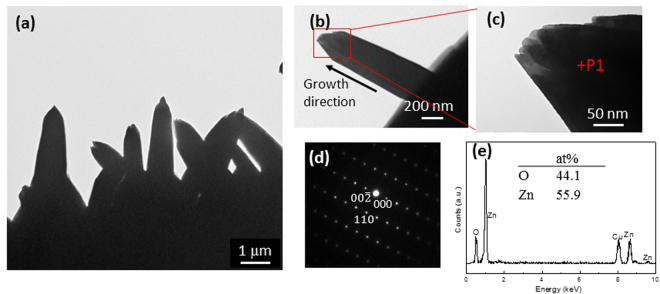
TEM images, SAED pattern, and EDS profile of the NRs after 24 h of UV irradiation on a copper mesh. (**a**), (**b**), and (**c**) show the TEM micrographs. (**d**) SAED pattern of P1 position in (**c**). (**e**) EDS profile of the ZnO NRs, and the inset shows atom ratio of O and Zn.

TEM observations of the ZnO NRs after 24 h of UV irradiation are shown in Fig. 4. The NRs have a tapered top with lengths in the range of 1–4 μm. The SAED pattern (Fig. 4d) was obtained along the $[1\bar{1}0]$ direction, and the growth direction is along the *c*-axis, with a $(00\bar{1})$ O-terminated polar surface, which is in accordance with previous reports^30,37^. The ratios of O and Zn were obtained from their electron diffraction spectroscopy (EDS) profile, and the results are shown in the inset table in Fig. 4e. As shown in the table, the ratio of O is slightly lower than that of Zn, which presumably results from the presence of oxygen vacancies^30^.

**Figure 5:**
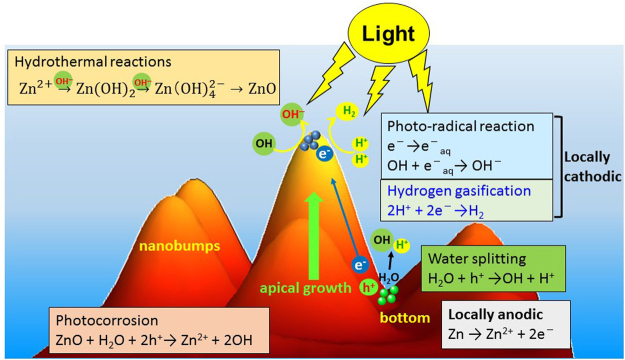
A schematic view of the mechanism of ZnO crystal growth in the SPSC process.

According to the above results, the photochemical reactions that occur during SPSC were deduced and a schematic illustration of the NC growth reaction mechanism is shown in Fig. 5, in which both photochemical and hydrothermal reactions contribute to the SPSC process^21,30,31^. First, electrons and holes are generated by photosemiconducting reaction (1) when UV light hits the nanobumps^38^.

1 $${\rm{SC}}+hv\to \mathrm{SC}\,({e}^{-}+{h}^{+})$$

The generated electrons build up at the apical portion of the nanobumps to generate a cathodic environment, whereas the holes left at the bottom of the concave nanobumps create a local anode^21,39^. Photochemical water-splitting reactions (reaction (2)) then build up holes at the bottom, which subsequently contribute to OH radical generation and to the photocorrosion of ZnO (reaction (3))^37,40^.

2 $${{\rm{H}}}_{2}{\rm{O}}+{h}^{+}\to {\rm{OH}}+{{\rm{H}}}^{+}\,({\rm{water}}\,{\rm{splitting}})$$

3 $${\rm{ZnO}}+{{\rm{H}}}_{2}{\rm{O}}+2{h}^{+}\to {{\rm{Zn}}}^{2+}+2{\rm{OH}}\,({\rm{photocorrosion}})$$

Meanwhile, electrons accumulated at the tip. The formation of hydrated electrons (reaction (4)) induces the transform of OH radicals to OH^−^ ions^41^ and contributes to the generation of an alkaline atmosphere at the end of nanobump tip^21^.

4 $${e}^{-}\to {e}_{aq}^{-}\,({\rm{hydrated}}\,{\rm{electron}}\,{\rm{formation}})$$

5 $${\rm{OH}}+{e}_{aq}^{-}\to {{\rm{OH}}}^{-}\,({{\rm{OH}}}^{-}\,{\rm{formation}})$$

Thus, the local separation of OH^−^ at the apex and H^+^ at the bottom occurs on the surface of the nanobumps (as shown in Fig. 5), which results in the apical growth of ZnO via hydrothermal reactions.

According to the pH change during the SPSC process, the formation of flower-like ZnO NCs can be divided into the following three reaction stages.

The first stage occurs immediately after submerging the specimen in water. As it was previously mentioned, the peak of pH curve was observed in the first several hours under both light and dark conditions; therefore, a corrosion micro-cell was assumed to form due to the nanobumps on the surface of the Zn plate, and allowing the anode and cathode reactions^42^ described below.

6 $${\rm{Zn}}\to {{\rm{Zn}}}^{2+}+2{e}^{-}\,({\rm{local}}\,{\rm{anode}}\,{\rm{reaction}})$$

7 $$2{{\rm{H}}}_{2}{\rm{O}}+2{e}^{-}\to {{\rm{H}}}_{2}+2{{\rm{OH}}}^{-}\,({\rm{local}}\,{\rm{cathode}}\,{\rm{reaction}})$$

OH^−^ generated by reaction (7) disperses in water and increases the water pH. At the same time, photoinduced reaction (3) also generates OH^−^ near the surface of the nanobumps through reaction (5).

Moreover, hydrogen gas generation was confirmed by gas chromatography (GC-14B, Shimadzu, Japan), as shown in Figure S2 in the Supplementary information.

The photocorrosion of ZnO (reaction (3)) also occurs at this stage. Along with these reactions, the pH temporarily increases for approximately 1–2 h (Fig. 1a). Through the OH^−^ generated by reactions (5) and (7), the following hydrothermal reaction to generate zinc hydroxide occurs, and the pH decreases.

8 $${{\rm{Zn}}}^{2+}+2{{\rm{OH}}}^{-}\,\to \,{\rm{Zn}}{({\rm{OH}})}_{2}$$

Then, the reaction of Zn → Zn(OH)_2_ can be written as follows:

9 $${\rm{Zn}}+2{{\rm{H}}}_{2}{\rm{O}}\to {\rm{Zn}}{({\rm{OH}})}_{2}+{{\rm{H}}}_{2}$$

In the second stage, after 6–10 h of UV irradiation, the pH slightly increases (Fig. 1a). The OH^−^ generated by reactions (5) and (7) causes the pH increase to alkaline levels, thereby generating the zinc hydroxide complex ion^40,43^ as shown below.

10 $${\rm{Zn}}{({\rm{OH}})}_{2}+2{{\rm{OH}}}^{-}\to {\rm{Zn}}{({\rm{OH}})}_{4}^{2-}$$

In the third stage, the following ZnO crystallization reaction occurs in alkaline solution^43^.

11 $${\rm{Zn}}{({\rm{OH}})}_{4}^{2-}\to {\rm{ZnO}}+2{{\rm{OH}}}^{-}+{{\rm{H}}}_{2}{\rm{O}}$$

Accordingly, the net reaction of ZnO growth via SPSC is represented as follows:

12 $${\rm{Zn}}+{{\rm{H}}}_{2}{\rm{O}}+hv\to {\rm{ZnO}}+{{\rm{H}}}_{2}({\rm{g}})$$

Thus, ZnO NRs were formed on the Zn substrate, along with hydrogen gas generation, via the SPSC process.

Because of the aforementioned local separation of OH^−^ at the apical and H^+^ at the bottom on the surface of the nanobumps/NRs by light-driven reactions^30^, synthesis of ZnO occurs by the hydrothermal reactions between Zn^2+^ and OH^−^ at the tip of the NRs. Therefore, ZnO NRs show an apical crystal growth.

**Figure 6:**
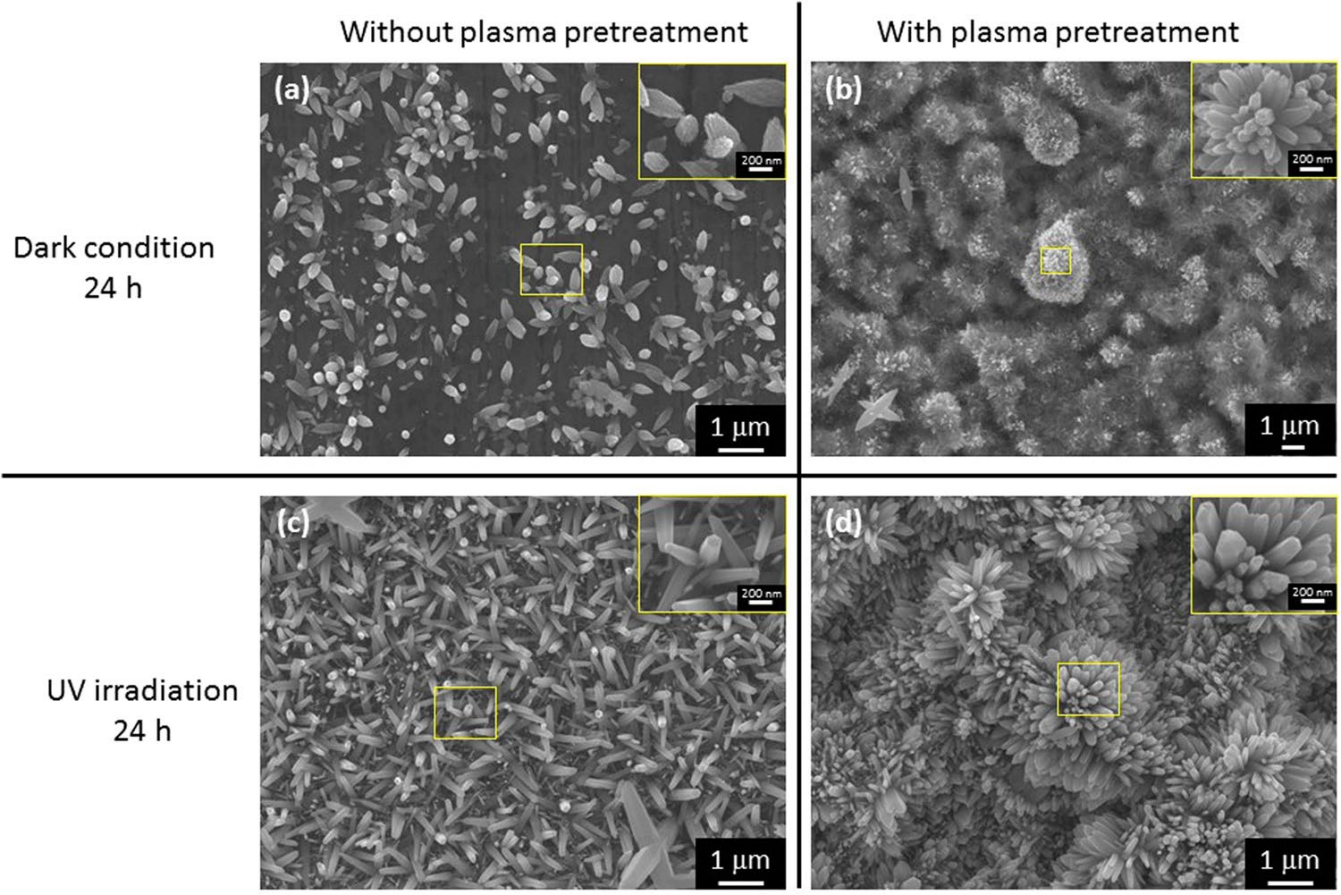
SEM images of the Zn plate: (**a**) untreated specimen in dark conditions for 24 h, (**b**) plasma-pretreated specimen in dark conditions for 24 h, (**c**) untreated specimen after UV irradiation for 24 h, and (**d**) plasma-pretreated specimen after UV irradiation for 24 h.

Furthermore, based on thermodynamic calculations using the HSC Chemistry software (Outokumpu Research Oy, Pori, Finland), ZnO formation can occur without illumination at room temperature because the Gibbs free energies (ΔG) of reactions (9) and (12) are approximately −79 kJ and −84 kJ, respectively. We obtained similar ZnO morphologies from the dark condition experiment. Figure 6 shows a comparison of the morphology of the surface of the Zn plates under dark conditions and under UV irradiation for 24 h. Two kinds of Zn plates, untreated specimens and plasma-pretreated specimens, were used. NRs were found on the surface of the untreated specimens under both dark and UV irradiation conditions (Fig. 6a,c). Comparing the morphologies of the two specimens, the NRs formed by UV irradiation have faceted characteristics, whereas those formed under dark conditions have a leaf-like structure. By contrast, when the specimen undergoes the plasma pretreatment process, the morphologies obtained under the two conditions are very different, and flower-like structures were observed. These results show that the plasma pretreatment process produced the aforementioned protrusions on the surface of the specimen, making the fabrication of flower-like structures easy. Additionally, in Fig. 6b, most of the specimen surface is covered with a dark fibrous film, which was detected by XPS as zinc hydroxide Zn(OH)_2_. Therefore, UV light greatly affects the NC morphology.

**Figure 7:**
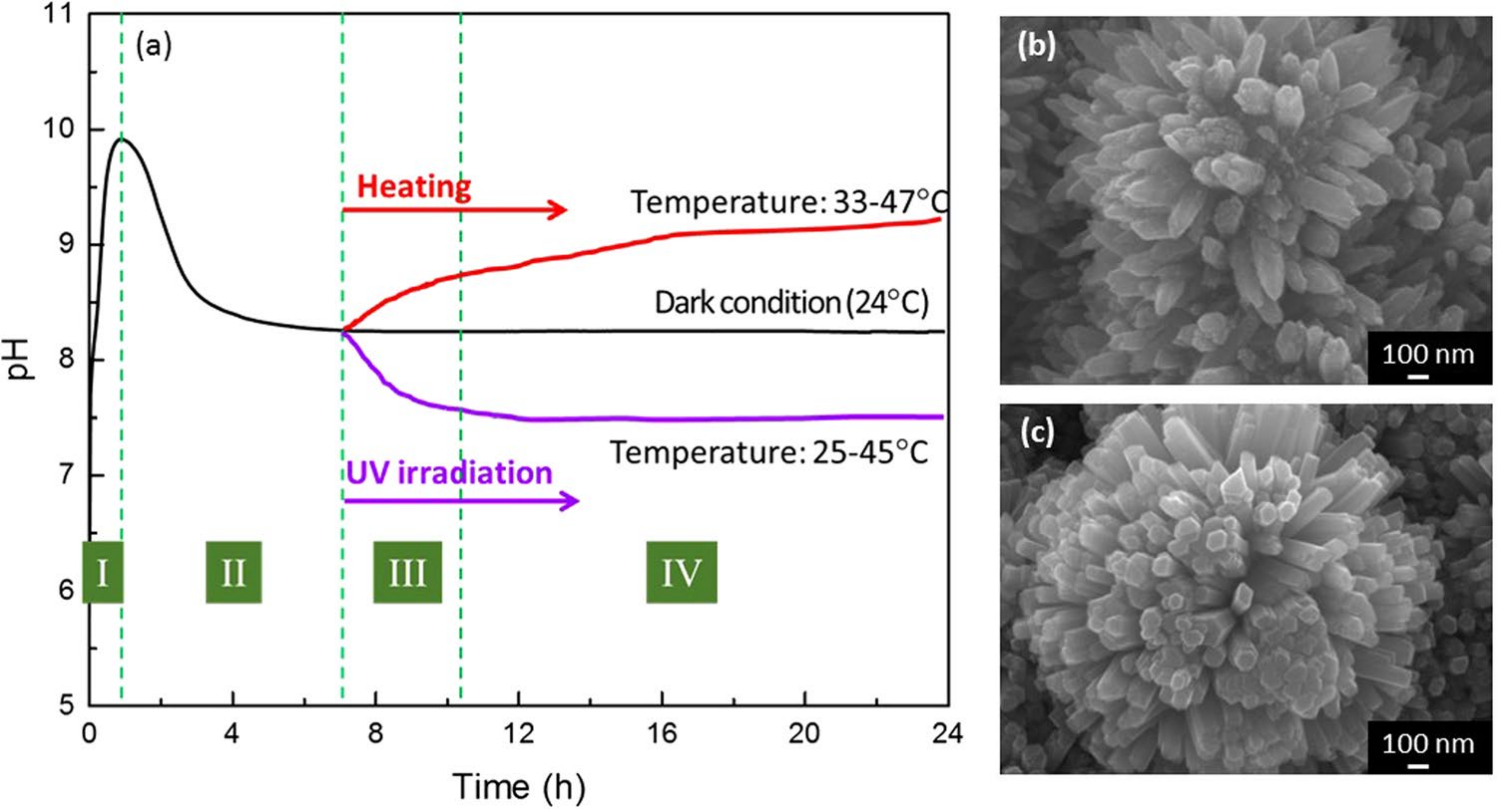
(**a**) Controlled experiment examining the pH change due to UV illumination or heating. (**b**) Morphology of the specimen after 7 h in dark conditions followed by 17 h of heating. (**c**) Morphology of the specimen after 7 h in dark conditions followed by 17 h under UV irradiation.

These results raise the following question: as NCs can be obtained under dark condition, what is the effect of UV irradiation on ZnO NCs, and what is the role of light irradiation in SPSC? To solve this question, additional controlled experiments were conducted. Plasma-pretreated specimens in ultrapure water were placed in different lightproof chambers. After the pH of the water stabilized, some of the submerged specimens were heated to 33–47 °C by a heater, and some were irradiated by UV light of different intensities. The pH curves of these experiments are shown in Figure S3 in the Supplementary information. Typical changes in the pH of water in these experiments are illustrated in the graph in Fig. 7a. The pH curves are divided into five stages. At room temperature and under dark conditions, the pH increased during the initial 2–4 h (stage I) and then decreased (stage II), which is similar to the results presented in Fig. 1a. After the initial sharp peak, the pH remained almost constant, and the water was slightly alkaline. In stage III, the pH and temperature changed upon UV irradiation or heating. If the water was heated to 33–47 °C under dark conditions, the pH tended to increase. By contrast, if the submerged specimens were exposed to UV light after the pH stabilized, the pH of the water decreased and trended toward neutral. At stage IV, the water temperature was stable.

The pH measured in the experiments reflect the OH^−^ concentration in the water. The photoinduced reaction formed OH^−^ on the NR surface, which mostly aggregated near the tip of the NRs^30^, whereas the pH of the medium water was relatively low. Additionally, the balance between ZnO growth and corrosion also affects the OH^−^ concentration in the water. Hydrothermal reactions (8), (10) and (11) can be written as the following net reaction (13):

13 $${{\rm{Zn}}}^{2+}+2{{\rm{OH}}}^{-}\leftrightarrow {\rm{ZnO}}+{{\rm{H}}}_{2}{\rm{O}}$$

Reaction (13) is a reversible reaction. The forward reaction shows the ZnO growth, and the backward reaction is the ZnO corrosion reaction. ZnO growth consumes OH^−^, and corrosion generates OH^−^. At thermodynamic equilibrium, the forward growth reaction rate is same as the backward corrosion reaction rate, and the OH^−^ concentration is stable, hence the pH of the water remains almost constant. In Fig. 7a, the pH is stable at the end of stage II, which shows the thermodynamic equilibrium of reaction (13). Upon increasing the water temperature by heating or UV irradiation (stage III in Fig. 7a), the change in environment breaks the thermodynamic equilibrium, which is reflected by the change in the pH of water. The increase in pH reflects that corrosion appears to dominate the reaction process. By contrast, a decrease in pH reflects that ZnO growth is dominant over corrosion. Thus, the change in pH under different conditions, as shown in Fig. 7a, shows that light irradiation makes ZnO growth dominant, and the pH of water decreases to near neutral, whereas the thermal energy from heating makes ZnO corrosion dominant, and the pH of water increases. The difference in the ZnO NC morphologies formed under the different conditions (Fig. 7b,c) shows that the NRs formed under UV light have faceted characteristics, whereas the NRs formed by heating the water under dark conditions show a corroded surface, which is in accordance with the above analysis.

**Figure 8:**
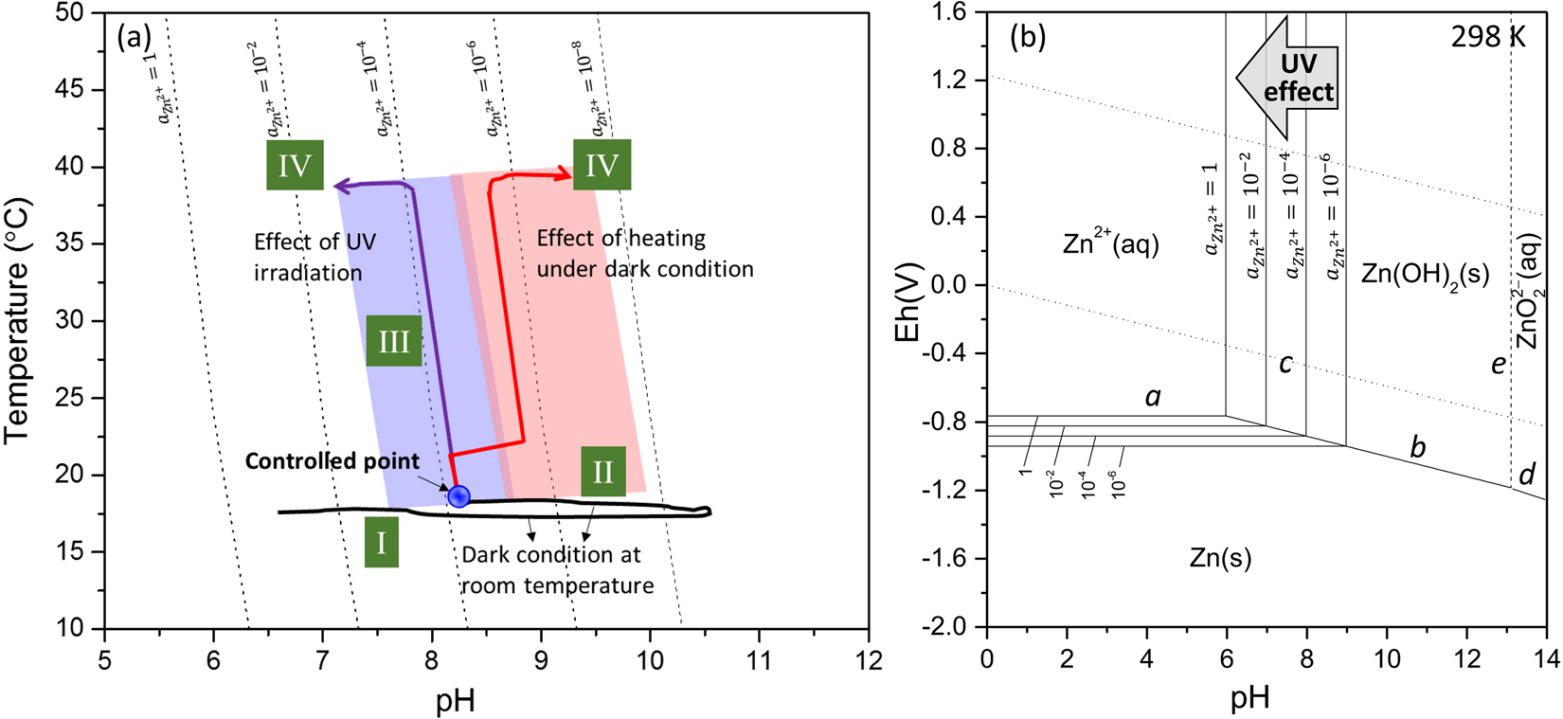
(**a**) Relation between pH and temperature for different experimental conditions. (**b**) Pourbaix diagram of the Zn/H_2_O system at 298 K^32^.

Moreover, the pH dependence of the water temperature in the controlled experiments is summarized as pH-T curves in Figure S4a and b, in the Supplementary information. The pH-T curves in the two figures have similar shapes. Consequently, their significant features are highlighted in Fig. 8a. When the pH is in the range of 7–11, reaction (8), ${{\rm{Zn}}}^{2+}({\rm{aq}})\leftrightarrow {\rm{Zn}}{({\rm{OH}})}_{2}({\rm{s}})$, occurs according to the Pourbaix diagram of the Zn/H_2_O system^32^ (Fig. 8b). The pH-T relations of reaction (8) are also given by the dotted lines in Fig. 8a, as determined from thermodynamic calculations of different Zn^2+^ activities in water (the calculation is shown in the Supplementary information). As shown in the figure, the pH-T curves can be divided into four stages, which correspond with the stages in Fig. 7a. Stage I and II represent the stage at which the pH increases and decreases, respectively, similar to the pH peak in Fig. 7a, when the specimen was submerged in ultrapure water in dark conditions at room temperature. After the pH returns to a steady state, UV irradiation or heating under dark conditions will propel the pH-T curve to stage III. In stage III, the temperature sharply increases, while at the same time, the pH slightly decreases, and the curves of stage III are approximately parallel to the calculated dotted line. In stage IV, the water temperature is relatively stable, whereas the pH values of the experiments change in opposite directions.

The relation between the pH and temperature for the reaction ${{\rm{Zn}}}^{2+}({\rm{aq}})\leftrightarrow {\rm{Zn}}{({\rm{OH}})}_{2}({\rm{s}})$ in thermodynamic equilibrium can be written as followings (the calculation is shown in the Supplementary information):

14 $${\rm{T}}=\frac{({{\rm{E}}}_{{\rm{Zn}}/{\rm{Zn}}{({\rm{OH}})}_{2}}^{0}-{{\rm{E}}}_{{\rm{Zn}}/{{\rm{Zn}}}^{2+}}^{0})\times 2{\rm{F}}}{2.3{\rm{R}}(2{\rm{pH}}+log{{\rm{a}}}_{{{\rm{Zn}}}^{2+}})}$$

where ${{\rm{E}}}_{{\rm{Zn}}/{{\rm{Zn}}}^{2+}}^{0}$ is the standard electrode potential of the reaction ${{\rm{Zn}}}^{2+}+2{e}^{-}\leftrightarrow {\rm{Zn}}$, −0.763 V; ${{\rm{E}}}_{{\rm{Zn}}/{\rm{Zn}}{({\rm{OH}})}_{2}}^{0}$ is the standard electrode potential of the reaction ${\rm{Zn}}{({\rm{OH}})}_{2}+2{{\rm{H}}}^{+}+2{e}^{-}\leftrightarrow {\rm{Zn}}+2{{\rm{H}}}_{2}{\rm{O}}$, −0.410 V; R is the universal gas constant, 8.314 J⋅mol^−1^⋅K^−1^; and F is the Faraday constant 96485 C⋅mol^−1^.

The above pH-temperature relation of the reaction ${{\rm{Zn}}}^{2+}({\rm{aq}})\leftrightarrow {\rm{Zn}}{({\rm{OH}})}_{2}({\rm{s}})$ can be simplified as

15 $${\rm{T}}=\frac{{\rm{C}}}{{\rm{pH}}+\alpha }$$

where ${\rm{C}}=\frac{({{\rm{E}}}_{{\rm{Zn}}/{\rm{Zn}}{({\rm{OH}})}_{2}}^{0}-{{\rm{E}}}_{{\rm{Zn}}/{{\rm{Zn}}}^{2+}}^{0})\times {\rm{F}}}{2.3{\rm{R}}}\,\,$is a constant and $\alpha =\frac{1}{2}{{\rm{loga}}}_{{{\rm{Zn}}}^{2+}}$, which is related to the Zn^2+^ activity.

Comparing the stage III and IV curves under the two conditions with the dotted lines in Fig. 8a, UV irradiation and heating greatly influenced the stage III and IV of pH-T curves: the former shows an increase in the Zn^2+^ activity, whereas the latter shows a decrease in the Zn^2+^ activity. The detail data of ${{\rm{a}}}_{{{\rm{Zn}}}^{2+}}$ could be obtained from equation (14) by experiment data of temperature and pH. Under UV irradiation condition, ${{\rm{a}}}_{{{\rm{Zn}}}^{2+}}$ is in the range of 10^−5.0^–10^−2.9^, and *α* is in the range of −2.5 to −1.5. Under dark condition, ${{\rm{a}}}_{{{\rm{Zn}}}^{2+}}$ is in the range of 10^−5.0^–10^−2.9^, and *α* is in the range of −3.6 to −2.0. Equation (15) also shows that the reaction temperature under UV irradiation is lower than that under dark conditions at the same pH condition because of the larger *α* value by UV irradiation.

To examine the different influences of UV irradiation and heating in dark conditions on the Zn^2+^ activity, the factor α can be rewritten as $\alpha =\frac{1}{2}{{\rm{loga}}}_{{{\rm{Zn}}}^{2+}}^{0}+\beta$, where ${{\rm{a}}}_{{{\rm{Zn}}}^{2+}}^{0}$ is the initial Zn^2+^ activity before the start of stage III (the Zn^2+^ activity at controlled point in Fig. 8a), which is at a steady state. *β* is a factor influenced by UV irradiation or heating. Under UV irradiation, the Zn^2+^ activity is almost constant in stage III and slightly increases in stage IV, and the factor *β* is a positive value and changes in the range of 0.2 to 0.5 in the present experiments. By contrast, under dark conditions, as the water temperature increases upon heating, the Zn^2+^ activity greatly decreases in stages III and IV; thus, the factor *β* is a negative value and changes in the range of −1.1 to −1.3 in this work. Therefore, UV irradiation and heating under dark conditions oppositely affect *β*. Because *β* increased with UV irradiation, we define *β* as the photoinduced enhancement factor.

Thermodynamically, the increased Zn^2+^ activity can enhance the ${{\rm{Zn}}}^{2+}\leftrightarrow {\rm{Zn}}{({\rm{OH}})}_{2}$ reaction, which indicates that both ZnO growth and corrosion can be activated by UV irradiation. As mentioned above, ZnO corrosion and growth occur at different positions on the nanobumps/NRs upon UV irradiation. The enhancement in these reactions by UV irradiation could enhance the apical growth of the NRs. Additionally, according to the Pourbaix diagram of Zn/H_2_O system^32^, both increasing the Zn^2+^ activity and increasing the system temperature can move line *c* in Fig. 8b to the left. Therefore, UV irradiation could allow the ${{\rm{Zn}}}^{2+}\leftrightarrow {\rm{Zn}}{({\rm{OH}})}_{2}$ reaction to occur at lower pH by increasing the ${{\rm{Zn}}}^{2+}$ activity, as shown by the direction of the arrow in Fig. 8b, without greatly increasing the system temperature. Thus, from the view point of pH and temperature changes of water in the SPSC process, UV irradiation could enhance the photocorrosion of ZnO and induce the apical growth of NRs by increasing the Zn^2+^ activity at relatively low temperature by preventing an increase in the pH of water, to sustain a neutral environment. This result reveals the environmentally friendly characteristics of the SPSC process.

**Figure 9:**
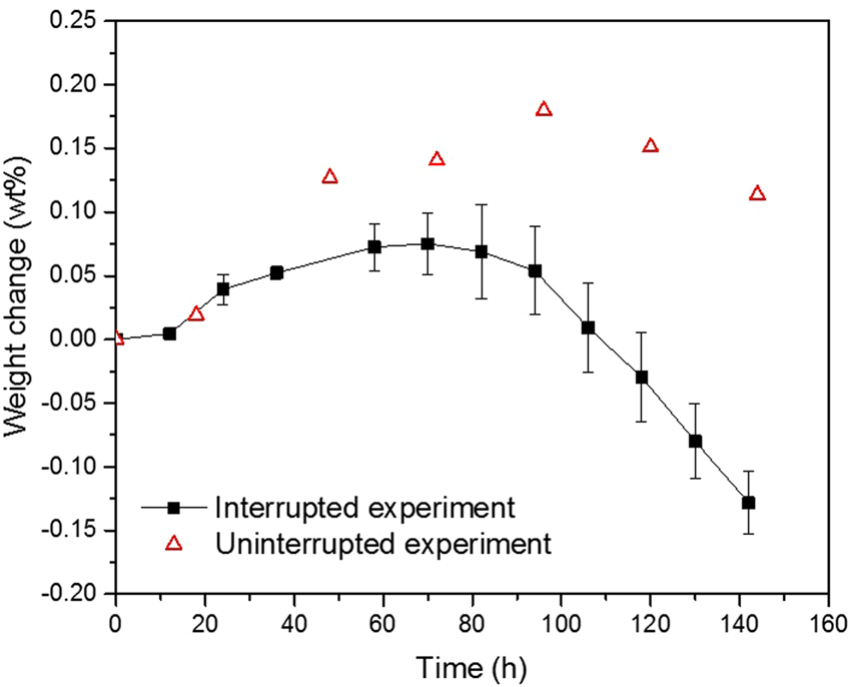
Weight change of the Zn plates during UV irradiation.

For the above reason, we observed the growth of ZnO NCs along the *c*-axis with respect to the UV irradiation time, as shown in the SEM images in Fig. 1. However, when the UV irradiation time was longer than 72 h, further ZnO growth was not observed. (Figure S5, Supplementary information). Accordingly, the NC growth was examined by evaluating the weight change of the specimens. Figure 9 shows the weight change of the Zn plate during UV irradiation. The black line is the average value from the interrupted experiment. For both the interrupted and uninterrupted experiments, the weight of the samples increased with UV irradiation time during the initial 60–96 h in increments of less than 0.2 wt%. Most of the interrupted specimens show a lower weight increment compared with the uninterrupted specimens. This effect was caused by the delamination of the NCs from the substrate Zn plate during the removal of the samples every several hours. During the stage at which the weight increased, the NC growth reaction was dominant. However, the weight increase of all the samples did not persist, and the weight decreased after 60–96 h of UV irradiation. After 144 h of UV irradiation, many exfoliated NCs were observed on the specimen, and flower-like ZnO NCs were no longer present (Figure S5, Supplementary information). In a previous report^30^, oxygen vacancy point defects were found to exist near the tip-edge of the NRs, and the ratio of O to Zn in the NRs increased with UV irradiation time. After 72 h of UV irradiation, the tip of NRs had a flat, capped shape, and the ratio of O to Zn was very close to 1. Therefore, the ZnO growth in the apical direction is dominant compared with ZnO photocorrosion until 72 h of UV irradiation. After that, further UV irradiation makes the ZnO corrosion reaction dominant on both the tip and bottom of the NRs, which result in the NCs having a corroded surface (Figure S5, Supplementary information). Further photocorrosion by UV irradiation leads to the exfoliation of NCs from the substrate Zn plate (Figure S5b,c, Supplementary information).

**Figure 10:**
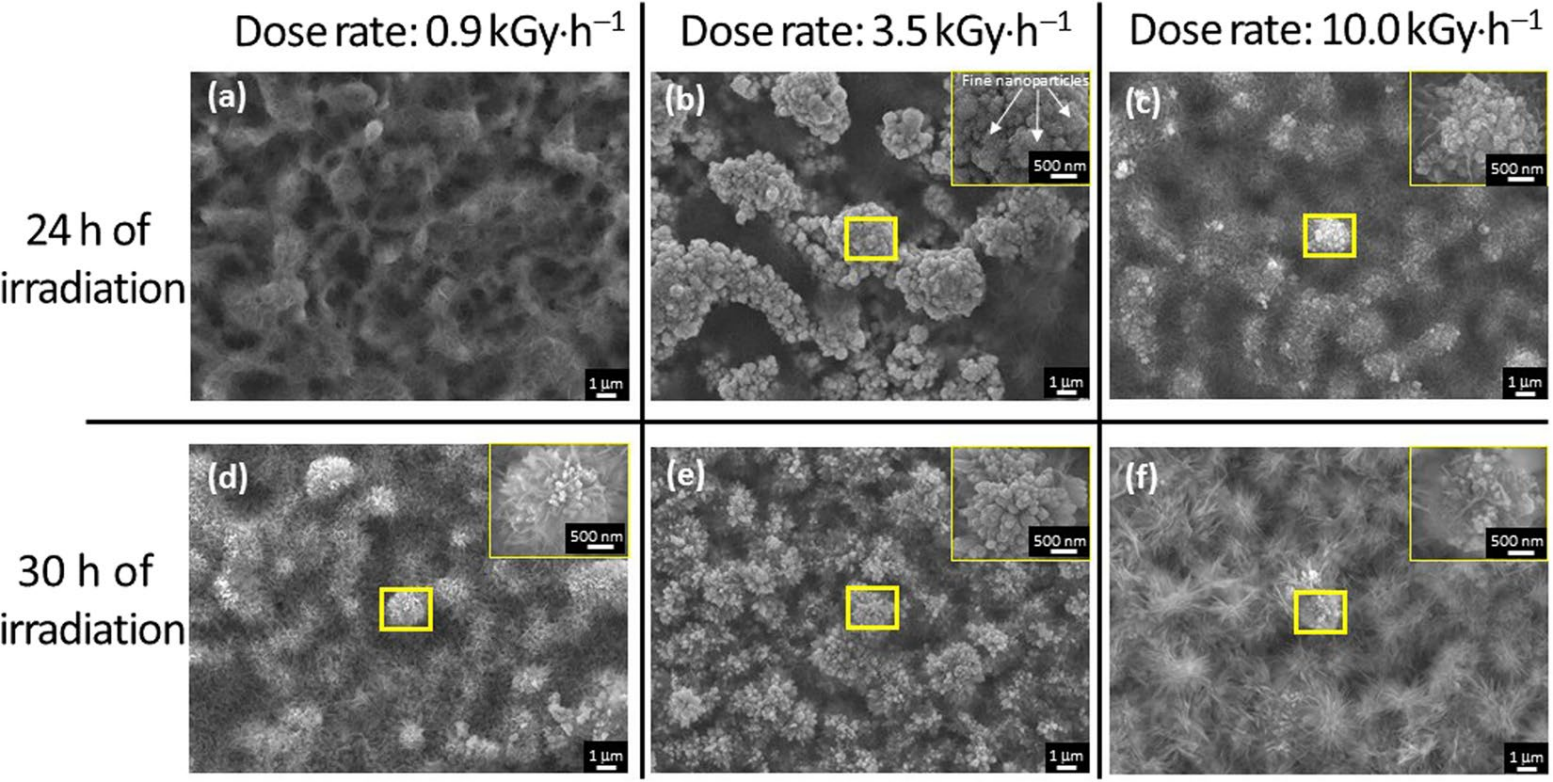
SEM images of the Zn plates after gamma-ray irradiation under different dose rates and irradiation times.

Based on the above discussion, SPSC involves photoradical reactions, which is the main source of OH^−^ for ZnO apical growth. To elucidate such photoradical reactions in the SPSC process, two additional experiments of gamma-ray irradiation and SOD addition were conducted. Gamma-ray irradiation can efficiently generate radiolysis products of water, such as OH, e^−^_aq,_ H(H_2_), H_2_O_2_, and H_3_O^+^ ^44^. Figure 10 shows the morphologies of the Zn specimens in the gamma-ray irradiation SPSC experiment. The dose rate was in the range of 0.9–10.0 kGy·h^−1^, and the irradiation time was 24 h and 30 h. At a dose rate of 0.9 kGy·h^−1^, a dark fibrous Zn(OH)_2_ structure was observed on part of the surface of the specimen, and NRs were not observed after 24 h of irradiation. When the irradiation time was increased to 30 h, flower-like nanostructures grew from the Zn(OH)_2_ substrate. When the dose rate was increased to 3.5 kGy·h^−1^, fine nanoparticles could be observed after 24 h of irradiation, and flower-like NRs were generated within 30 h of irradiation. However, when the dose rate was increased to 10.0 kGy·h^−1^, most of the nanoparticles were covered with a dark fibrous Zn(OH)_2_ film after 24 h of irradiation. In addition, a large-area Zn(OH)_2_ layer over the NRs was also observed when the irradiation time was increased to 30 h (Fig. 10f) and 48 h (Figure S6a, Supplementary information) under 10.0 kGy·h^−1^, and the re-deposition of ZnO was not observed. Therefore, the Zn(OH)_2_ layer on the NRs restricted the further growth of ZnO nanoparticles. As mentioned earlier, when Zn(OH)_2_ formed on the tips of the NRs, reactions (9) and (10) for the process Zn(OH)_2_→${\rm{Zn}}{({\rm{OH}})}_{4}^{2-}$→ZnO occurred. In this case, a local alkaline environment near the nanobumps is needed. However, after gamma-ray irradiation condition, the pH of water was in the range of 4.1–6.6. Once the Zn(OH)_2_ layer formed over a relatively large area, the local OH^−^ concentration decreased, and the further formation of ${\rm{Zn}}{({\rm{OH}})}_{4}^{2-}$ was difficult. We speculate that this effect is the main reason for the restriction of the further growth of NRs. Therefore, under gamma-ray irradiation at a relatively lower dose rate, fine nanoparticles were obtained, and the nanoparticles grew with irradiation time. In general, fine crystal submerged synthesis can be induced by gamma rays with high linear-energy-transfer (LET) radiation.

**Figure 11:**
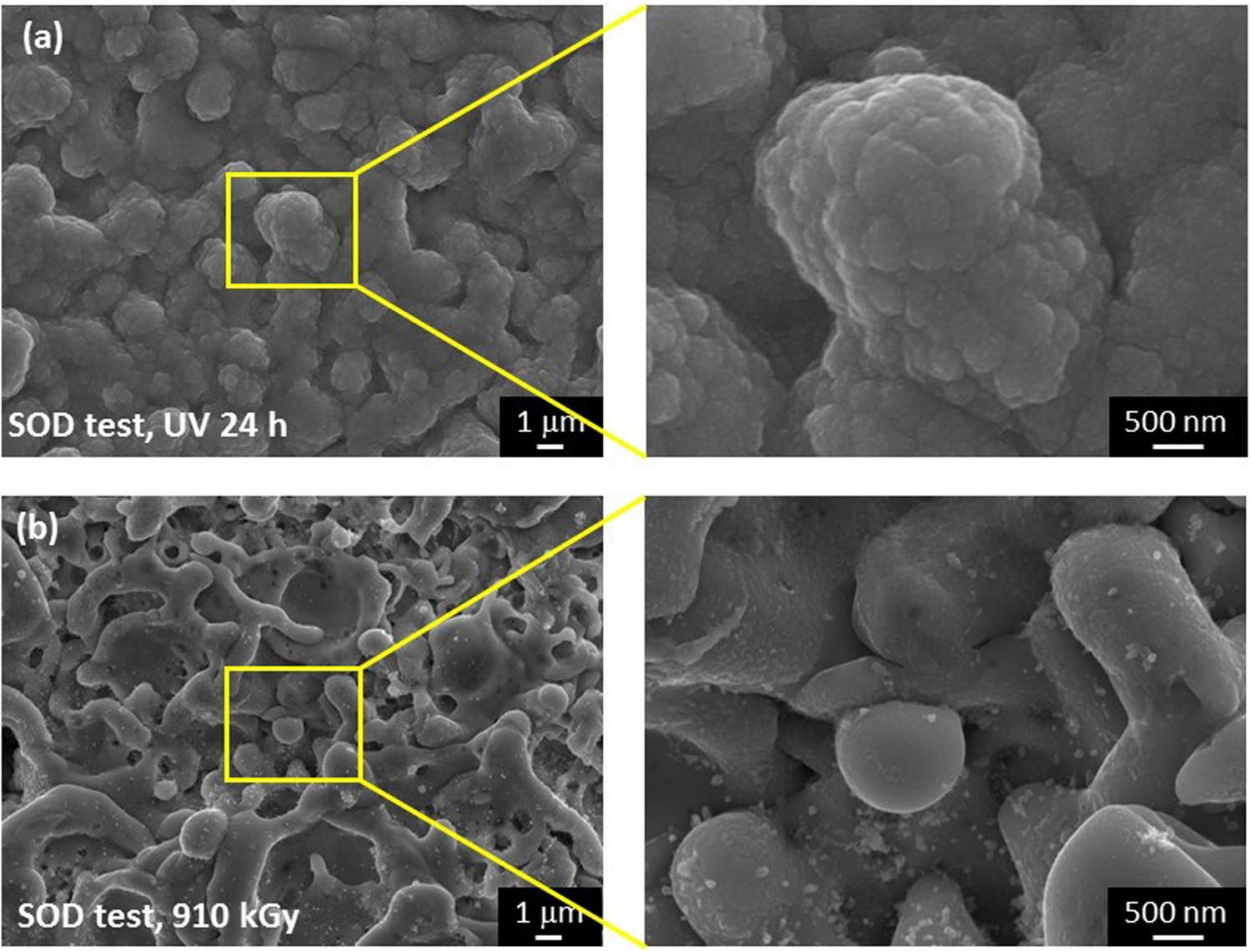
SEM images of the specimens with SOD addition: (**a**) specimen after 24 h of UV irradiation and (**b**) specimen after gamma-ray irradiation at a dose of 910 kGy (14 kGy·h^−1^, 65 h).

Additionally, Fig. 11 shows the SEM images of the specimen surfaces with SOD addition after UV (Fig. 11a) and gamma-ray irradiation (Fig. 11b). No NCs were found in either UV-irradiated specimen or the gamma-ray irradiated specimen with SOD addition. The SOD reagent is known to capture O_2_^−^ ^45,46^. Compared with the specimens without SOD reagent addition (Fig. 1e and Figure S6b in the Supplementary information), the specimens without SOD addition showed NCs formation on the surface, whereas the crystal formation was suppressed in the specimens with SOD addition. This result suggested that O_2_^−^ plays an important role in ZnO growth and revealed that crystal formation via SPSC was caused by photolysis or radiolysis in oxygenated water.

## Conclusion

The SPSC method, proposed as a new green technology that requires only water and light, can be applied to produce ZnO NCs. We carried out surface microstructural analysis and monitored the pH and temperature change of the water during the process. Based on this analysis, ZnO NCs formation mechanism was clarified. Furthermore, the effect of light irradiation during the process was elucidated by contrast experiments under dark conditions. It shows that both photoinduced reactions and hydrothermal reactions contribute to the SPSC process. The former generates OH radicals, which are one of the main sources of OH^−^ at the crystal tips, whereas the latter involve the ZnO growth reactions of Zn^2+^ → Zn(OH)_2_ → ${\rm{Zn}}{({\rm{OH}})}_{4}^{2-}$→ ZnO. Furthermore, ZnO NCs can be obtained under both light irradiation and heating in dark conditions, in which ZnO growth and corrosion reactions occur simultaneously. Light irradiation makes ZnO growth dominant and the water pH close to neutral, whereas thermal energy makes ZnO corrosion dominant and the water pH increases. The role of light in SPSC process is to enhance ZnO apical growth at relatively lower temperature by preventing the pH of water from increasing, revealing the environmentally benign characteristics of the present process.

## Methods

### Surface Pretreatment

Surface pretreatment was conducted in a submerged liquid plasma experiment device (shown in Figure S7a, Supplementary information). A platinum wire (length: 1000 mm, diameter: 0.5 mm, 99.98 mass%, Nilaco, Japan) served as the anode. The cathode comprised the zinc plate (35 × 5 × 0.5 mm, 99.5%, Nilaco, Japan) wired with copper wire (diameter: 0.5 mm, 99.99%, Nilaco, Japan). Voltage was applied using a direct current (DC) power supply (ZX800H, Takasago). The electrolyte was a 100 mol·m^−3^ K_2_CO_3_ solution. The electrolyte temperature was measured by a polymer-coated thermistor thermometer. The current and the voltage were measured by the DC power supply. The plasma treatment was carried out in the range of 130–140 V for 10 minutes. The pretreated Zn plates was washed by deionized water and then was cut to a length of 20 mm for subsequent SPSC experiment.

### SPSC Experiment

Two types of the SPSC experiments were conducted: UV irradiation and gamma-ray irradiation.

During the UV-irradiation SPSC experiment, a plasma-treated Zn plate was placed into a 4-mL cuvette with ultrapure water (Wako Pure Chemical, Japan), which was deaerated by boiling, and then irradiated by a UV lamp (UVP, B-100AP, USA, λ = 365 nm, 3.4 eV) for 0–144 h in a lightproof chamber (shown in Figure S7b, Supplementary information). The intensity of the UV irradiation was 10−53 mW·cm^−2^. During the SPSC experiment, the pH and temperature of the water were measured using a pH/ORP meter (Horiba, LAQUA, D-72) containing a micro ToupH electrode (Horiba, LAQUA, 9618 S) and a long ToupH electrode (Horiba, LAQUA, 9680S-10D).

In the gamma-ray irradiation SPSC experiment, the plasma-treated Zn plate was placed into a test tube with 3 mL ultrapure water and then irradiated with gamma-rays, which was performed at the ^60^Co irradiation facility of the Institute of Scientific and Industrial Research (ISIR) at Osaka University. The gamma-ray dose rate was determined by the distance from the sample to the ray source. The absorbed dose was calculated by Fricke dosimetry.

### Dark Condition Experiment and Superoxide Dismutase (SOD) Addition Experiment

The dark condition experiment was conducted in a lightproof chamber without illumination, in which the Zn plate was immersed in deaerated ultrapure water for several hours. In the dark condition experiment, a heater was used to control the water temperature. The SOD addition experiment was performed using both UV and gamma-ray irradiation SPSC experimental procedures. SOD from bovine erythrocytes (Sigma–Aldrich, USA) was dissolved in ultrapure water at a concentration of 1.4–3.0 g/100 mL.

### Weight Change Measurement

Two types of weight change measurements, interrupted and uninterrupted experiments, were conducted by a microbalance (AEM-5200, Shimadzu, Japan). The interrupted experiment involves the weight measurement of a sample at different UV irradiation times. The sample was removed from the cuvette for measurement after a certain period of UV irradiation and then returned to the cuvette to continue the irradiation. The uninterrupted experiment measures weight change of different samples UV irradiated for different times.

### Physical Characterization

X-ray diffraction (XRD) patterns of the samples were obtained using an X-ray diffractometer (Rigaku, Miniflex) equipped with a Cu K*α* source operating at 40 kV and 15 mA. The surface morphologies were observed by field emission scanning electron microscopy (FE-SEM, JSM-7001FA, JEOL). Transmission electron microscopy (TEM) and selected area electron diffraction (SAED) patterns for the crystal were obtained using a conventional transmission electron microscope (JEM-2000FX, JEOL) operated at 200 kV. The nanoparticles were characterized using an X-ray photoelectron spectroscopy (XPS, JEOL, JPS-9200), equipped with a monochromatic Al K*α* X-ray source (1486.6 eV). The analyzed area of the samples was 3 mm × 3 mm (large scale). The peak positions and areas were optimized by a weighted least-squares fitting method using 70% Gaussian and 30% Lorentzian line shapes. All XPS spectra were calibrated to the C (1 s) core level peak at 286.0 eV.

## Electronic supplementary material

Supplementary information
