# Supplementary material for: Photochemistry and the role of light during the submerged photosynthesis of zinc oxide nanorods
Source: Sci Rep. 2018 Jan 9;8:177. doi: 10.1038/s41598-017-18572-8 (PMC5760726; doi:10.1038/s41598-017-18572-8)
Supplement: Supplementary file 1 — Supplementary information [file 41598_2017_18572_MOESM1_ESM.pdf]

Supplementary information

Photochemistry and the role of light during the submerged photosynthesis of zinc oxide nanorods

Lihua Zhang[1], Melbert Jeem[2], Kazumasa Okamoto[1], and Seiichi Watanabe[1,*]

1. Faculty of Engineering, Hokkaido University, N13, W8, Kita-ku, Sapporo 060-8628, Japan.

2. Graduate School of Engineering, Hokkaido University, N13, W8, Kita-ku, Sapporo 060-8628, Japan.

Figure S1. SEM images of the specimens: (a) after plasma pretreatment, (b) after 1 h of UV irradiation.

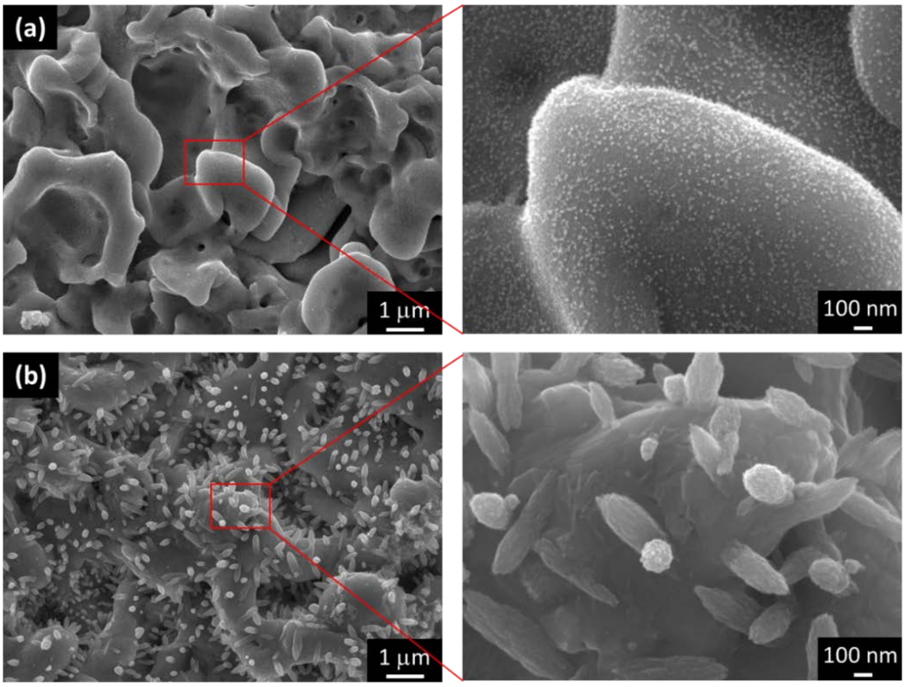

Figure S2. Chromatogram of the gas collected from the SPSC process. Three peaks corresponding to H_2, O_2, and N_2 were detected. The inset shows the magnified view of the O_2 and N_2 peaks. The area ratio of the O_2 peak to the N_2 peak in the figure is same as the result measured for air. Therefore, the O_2 and N_2 detected in the experiment are from entrained air during gas collection, only H_2 gas was generated during the SPSC process.

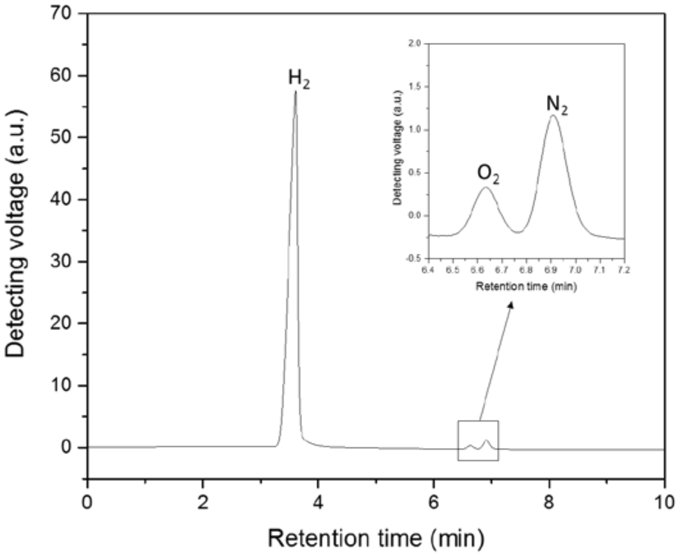

Figure S3. Time dependence of the pH and temperature of ultrapure water during the controlled experiments: (a) under dark conditions at room temperature (24°C), (b) under dark conditions and heating the water to approximately 35°C at the half way point, (c) under dark conditions and heating the water to approximately 42°C at the half way point, (d) under dark conditions at 12°C with UV irradiation (intensity: 53 mW·cm^{-2}) at the half way point, (e) under dark condition at 12°C with UV irradiation (intensity: 28 mW·cm^{-2}) at the half way point, and (f) under dark condition at 23°C with UV irradiation (intensity: 28 mW·cm^{-2}) at the half way point.

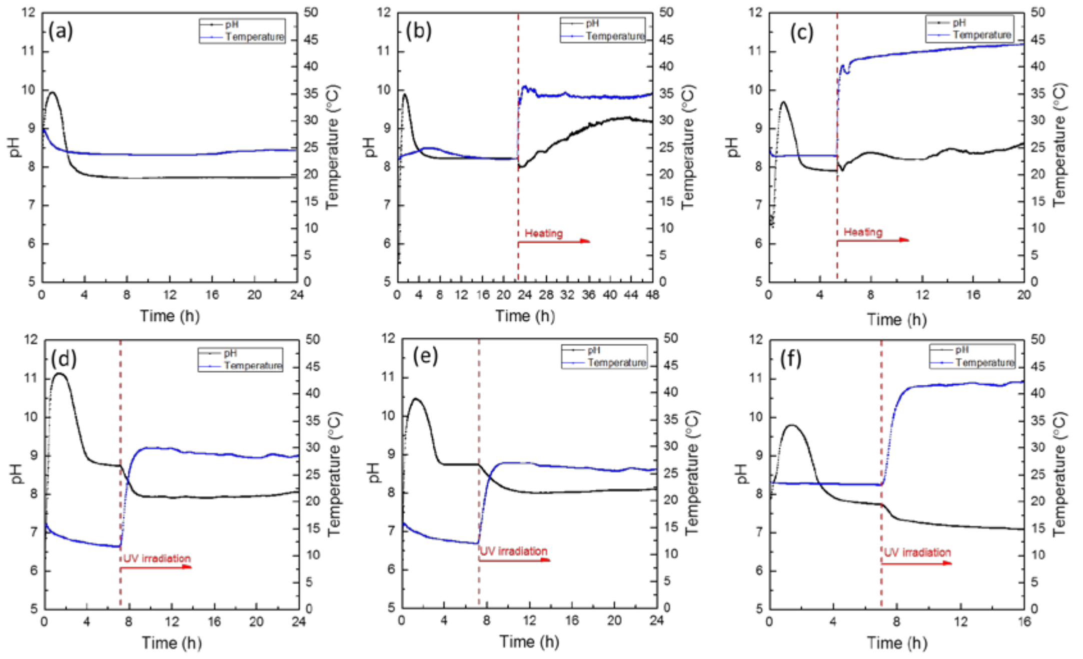

Figure S4. The relation between pH and temperature of ultrapure water during the controlled experiments: (a) under dark conditions with UV irradiation at the half way point and (b) under dark conditions with heating at the half way point.

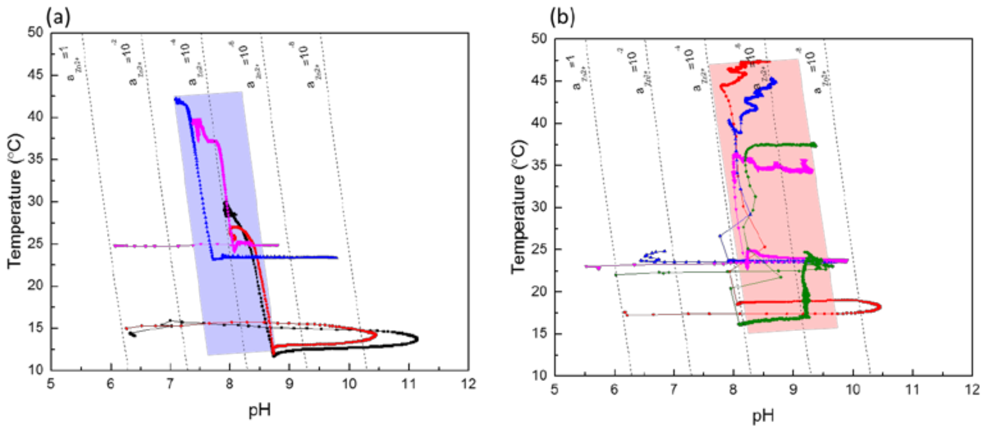

Figure S5. SEM images of the specimens after different UV irradiation times: (a) 96 h, (b) 120 h, and (c) 144 h.

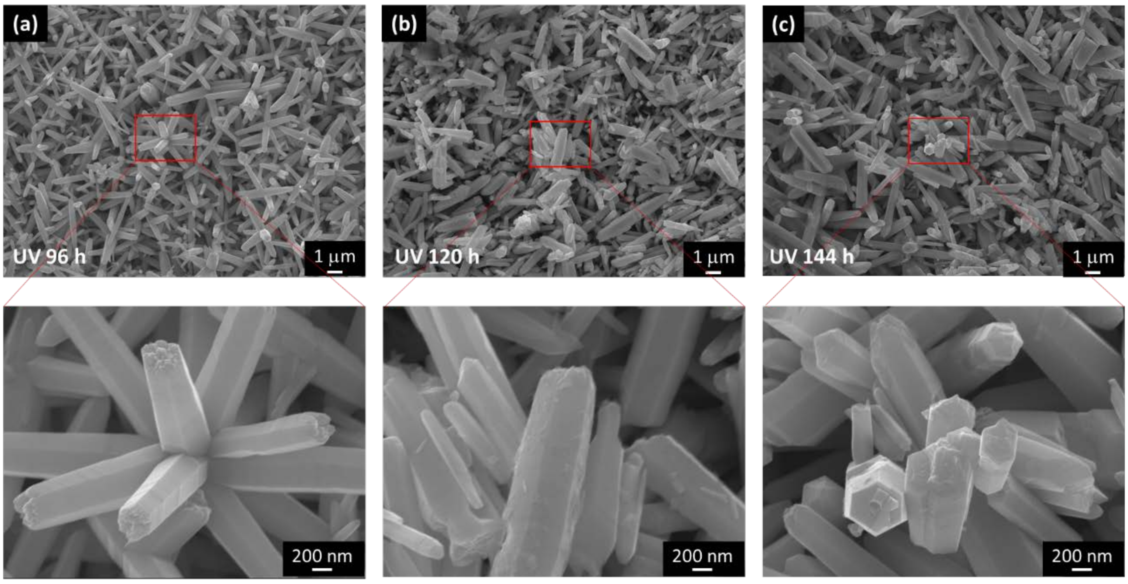

Figure S6. SEM images of the specimens after gamma-ray irradiation at different dose rates and irradiation times: (a) 10.0 $kGy \cdot h^{-1}$, 48 h and (b) 14.0 $kGy \cdot h^{-1}$, 65 h.

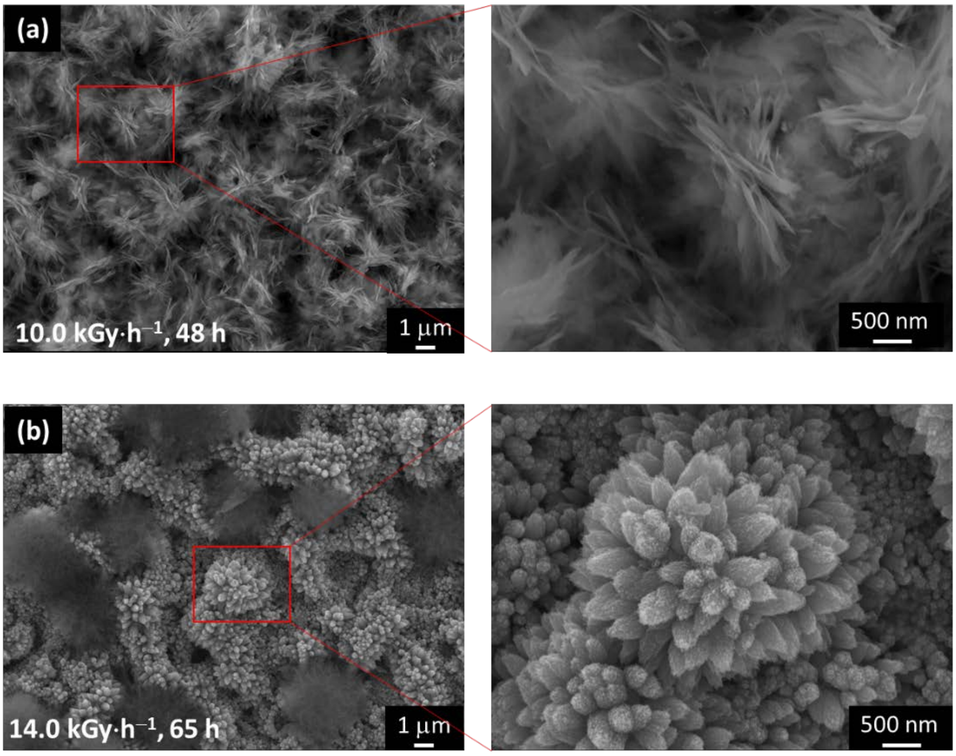

Figure S7. Schematic diagrams of the (a) plasma pretreatment experiment and (b) UV-irradiation SPSC experiment.

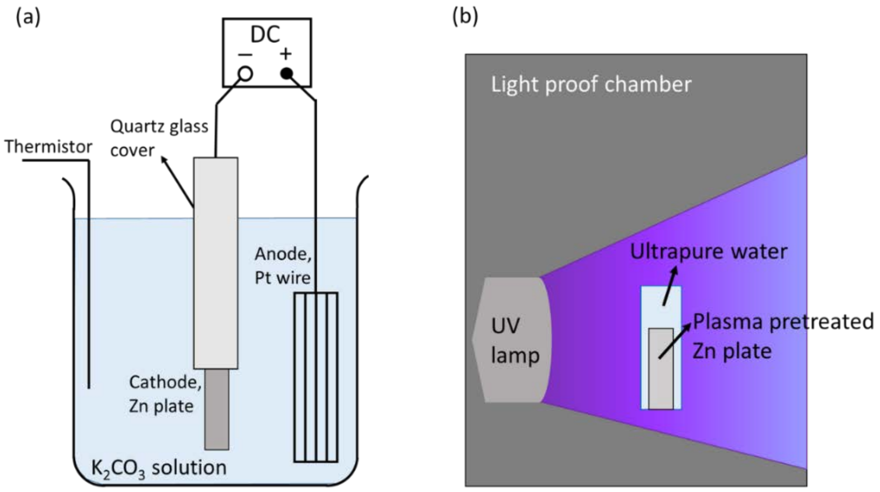

Supplementary materials for the calculation of dotted lines in Figure 8a and equation (14):

In Figure 8b, three reactions involving Zn, Zn^{2+}, and $Zn(OH)_2$ are presented here. The reactions for lines *a*, *b* and *c* in the figure are as follows:

$$\mathrm{Zn}^{2+} + 2e^- \leftrightarrow \mathrm{Zn} \qquad \text{(SI – 1)}$$

$$Zn(OH)_2 + 2H^+ + 2e^- \leftrightarrow Zn + 2H_2O \qquad \text{(SI – 2)}$$

$$Zn^{2+} + 2OH^- \leftrightarrow Zn(OH)_2 \qquad \text{(SI – 3)}$$

According to the Nernst equation, the equilibrium potential of reactions (SI – 1) and (SI – 2) are as follows:

$$E_{eq,a} = E^0_{Zn/Zn^{2+}} - \frac{RT}{2F} ln \frac{a_{Zn}}{a_{Zn^{2+}}} \qquad \text{(SI – 4)}$$

$$E_{eq,b} = E^0_{Zn/Zn(OH)_2} - \frac{RT}{2F} ln \frac{a_{Zn}}{a_{Zn(OH)_2} a^2_{H^+}} \qquad \text{(SI – 5)}$$

where $E^0_{Zn/Zn^{2+}}$ and $E^0_{Zn/Zn(OH)_2}$ are the standard electrode potential; R is the universal gas constant, 8.314 J·mol^{-1}·K^{-1}; and F is the Faraday constant 96485 C·mol^{-1}. As $\mathrm{pH} = -log a_{H^+}$, the pH of line *c* can be calculated when $E_{eq,a} = E_{eq,b}$:

$$\mathrm{pH} = \frac{(E^0_{\frac{Zn}{Zn(OH)_2}} - E^0_{Zn/Zn^{2+}})F}{2.3RT} - \frac{1}{2} log a_{Zn^{2+}} \qquad \text{(SI – 6)}$$

Hence, the relation between the temperature and pH can be rewritten as following equation, which is the same as the equation (14):

$$\mathrm{T} = \frac{(\mathrm{E}^0_{\mathrm{Zn/Zn(OH)_2}} - \mathrm{E}^0_{\mathrm{Zn/Zn^{2+}}}) \times 2\mathrm{F}}{2.3\mathrm{R}(2\mathrm{pH} + log a_{\mathrm{Zn^{2+}}})} \qquad \text{(SI – 7)}$$

In equation (SI – 7), if $a_{Zn^{2+}}$ is given, the curve of temperature and pH can be drawn. In Figure S4 and Figure 8a, the dotted lines show the relation of temperature and pH when $a_{Zn^{2+}}$ is 1, 10^{-2}, 10^{-4}, 10^{-6}, 10^{-8}, respectively.
